# Levonorgestrel intrauterine system embedded within tubal ectopic pregnancy: a case report

**DOI:** 10.1186/s13256-021-02723-7

**Published:** 2021-03-09

**Authors:** Dorothy Makena, Ingrid Gichere, Khadija Warfa

**Affiliations:** Department of Obstetrics and Gynecology, Aga Khan University Hospital Nairobi, P.O. Box 30270-00100, Nairobi, Kenya

**Keywords:** IUD, Mirena, Levonorgestrel IUS, Ectopic pregnancy, Tubal migration

## Abstract

**Background:**

The presence of the levonorgestrel-releasing intrauterine system embedded within an ectopic pregnancy is a rare occurrence. Tubal migration of an intrauterine device is not well understood and has not been extensively studied in literature.

**Case presentation:**

A 34-year-old African woman, para 1, gravida 2, presented with symptoms of ruptured ectopic pregnancy. She underwent a laparoscopy where a ruptured left ectopic pregnancy was found with a levonorgestrel-releasing intrauterine system inserted 2 years prior embedded within the tube. A left salpingectomy was performed with removal of the levonorgestrel-releasing intrauterine system. The patient recovered well and proceeded to have an intrauterine pregnancy 3 months later.

**Conclusion:**

Migration of the levonorgestrel-releasing intrauterine system into the fallopian tube is a rare occurrence that is not well understood. In the case presented, levonorgestrel-releasing intrauterine system was found embedded within the fimbrial end of the left fallopian tube, which had a ruptured ectopic pregnancy. Surgical treatment with laparoscopy is recommended for intraabdominal intrauterine device to prevent complications.

## Background

An intrauterine contraceptive device (IUD) is a highly effective method of contraception [1]. Types of IUDs include the levonorgestrel releasing intrauterine system (LNG IUS), also commonly referred to as Mirena, and the copper IUD. They have a low failure rate of less than 1%, which is comparable to permanent sterilization [1]. The pearl index of the LNG IUS is 0.06 per 100 women years, while that of the copper IUD is 0.52 per 100 women years [2]. Even though the risk of ectopic pregnancy with IUDs is lower than with no contraception, if a pregnancy occurs with an IUD *in situ*, it is likely to be an ectopic pregnancy. The rate of ectopic pregnancy among LNG IUS users ranges from 0.02 to 0.2 per 100 women years, whereas the rate among copper IUD users ranges from 0.1 to 0.8 per 100 women years. In case of pregnancy, the risk of ectopic pregnancy is higher in LNG IUS users compared with copper IUD users (27% *versus* 15% respectively) [2]. Progesterone is known to cause ciliary dysfunction within the fallopian tube, subsequently predisposing LNG IUS users who conceive to ectopic pregnancy [3]. It is therefore important to rule out ectopic pregnancy in women with acute abdomen or positive pregnancy test among LNG IUS users [4]

A correctly positioned IUD is located within 3 mm of the uterine fundus, with both arms extending to the cornua, a vertically oriented stem in the uterine body, and the strings protruding through the cervical os into the vaginal canal [5]. Suboptimally placed IUDs are at a higher risk of malposition or expulsion and associated symptoms [5]. Malposition of IUDs is a common complication of this method of contraception, with a reported rate of up to 10% [6]. Malposition of an IUD includes displacement, expulsion, rotation, or embedment. Migration of IUDs is, however, an uncommon occurrence, with most cases of migration reported to the colon and the urinary tract [7–9]. The fallopian tube is an uncommon location for migration and embedment [7]. Having an IUD embedded within a tubal ectopic pregnancy is an even rarer phenomenon.

We describe a case of a patient who presented with ruptured ectopic pregnancy and was found to have a LNG IUS embedded in the fimbrial end of the affected fallopian tube.

## Case presentation

A 34-year-old African female, para 1, gravida 2, presented to the Accident and Emergency Department, having had symptoms of vomiting and abdominal pain for 3 days. The symptoms had worsened on the day of presentation to the hospital. She reported several episodes of vomiting with associated loose stools and abdominal fullness. She also had ongoing vaginal bleeding that had started 5 days prior to presentation.

Two years prior, the patient had an uncomplicated insertion of LNG IUS by an obstetrician/gynecologist at the 8-week visit following a normal vaginal delivery. She had a normal pap smear done at the time of insertion. One year following insertion, she had a desire to conceive and was scheduled for removal of the LNG IUS device. The strings could not be seen, and the device could not be retrieved with alligator forceps. The patient was therefore sent for a pelvic ultrasound to locate the lost IUD. The device was not seen on ultrasound. The patient was, however, lost to follow-up until presentation with symptoms of ruptured ectopic pregnancy. She had no preexisting conditions or previous surgery.

On physical examination she was in fair general condition and not pale. Her vital signs were a temperature of 37.6 °C, a blood pressure of 120/66 mmHg, pulse rate of 99 beats per minute, respiration rate of 18 breaths per minute, and oxygen saturation of 100% on room air. On abdominal examination she had tenderness on the left iliac fossa and suprapubic regions with absent bowel sounds. The rest of the systemic examination was normal. An impression of acute abdomen was made at this point. As initial treatment she was given intravenous fluids (Ringer’s lactate solution) 1-L bolus, as well as intravenous paracetamol and ondansetron for pain and vomiting, respectively.

**Fig. 1:**
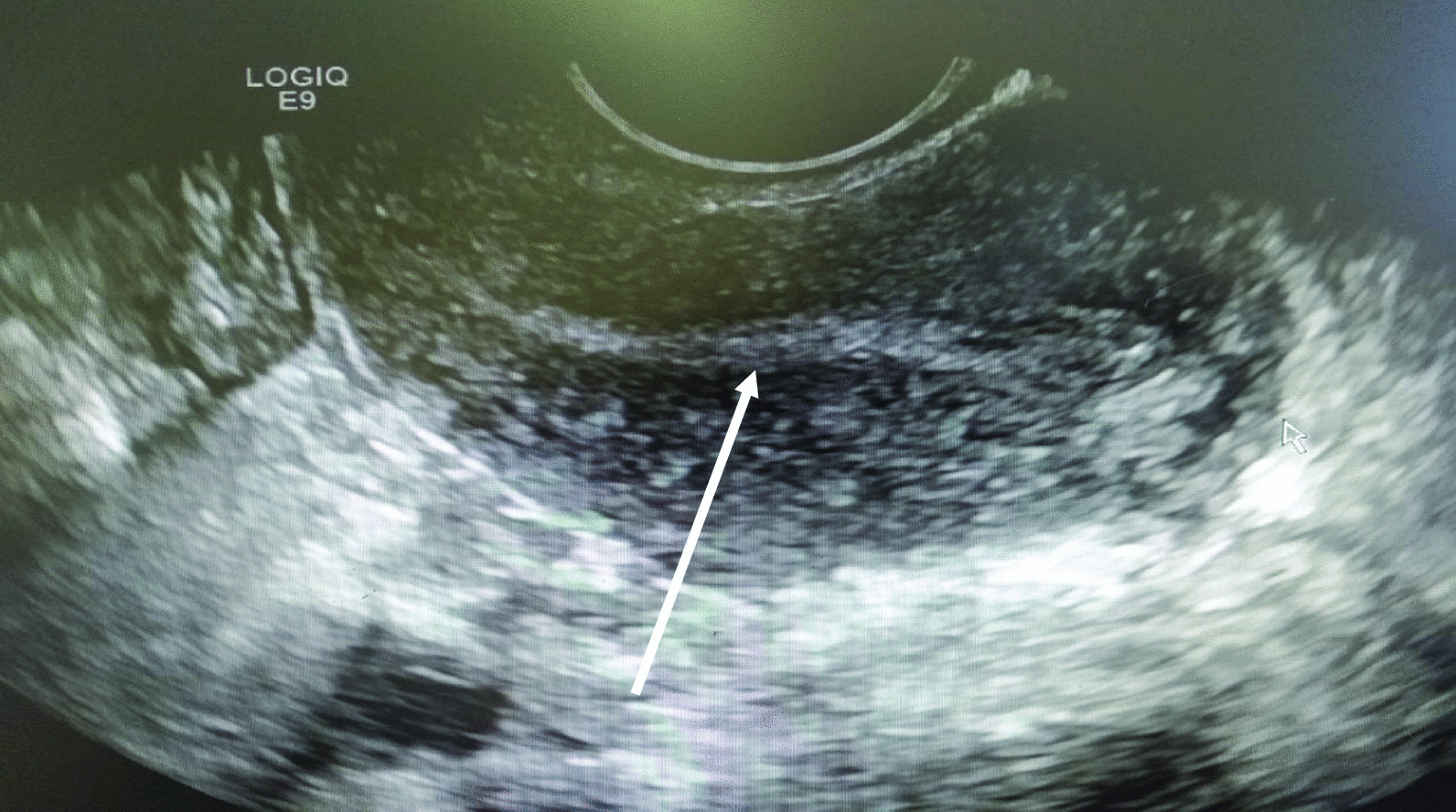
Ultrasound image of an empty uterus. The arrow points to the endometrial lining measuring 5.5 mm

The initial investigations included a full blood count, which revealed a normal hemoglobin level of 13.2 g/dl, slightly elevated white cell count of 12.28 × 10^9^ cells/L, and normal platelet count of 314 × 10^9^ cells/L. She had a beta human chorionic gonadotropin (Hcg) level of 7721 mIU/ml. Urinalysis showed leucocytes 2+, nitrite negative, and blood 2+. Transvaginal ultrasound showed a 2.1 cm × 1.8 cm echogenic mass with central cystic area on the left adnexa. It had no internal or peripheral vascularity. There was marked pelvic echogenic free fluid with low internal echoes extending to the Morrison’s pouch. The uterus was anteverted and normal in size and shape with an endometrial thickness of 5.5 mm. A 1.9 cm cystic lesion was seen in the right ovary, which was likely a corpus luteum cyst. There was no gestational sac or intrauterine device seen within the endometrial cavity (Fig. 1). These features indicated ruptured ectopic pregnancy.

The diagnosis at this point was a ruptured left tubal ectopic pregnancy. The plan was to admit the patient for an emergency laparoscopy with possible left salpingectomy. The diagnosis and plan were explained to the patient, who signed an informed consent for the procedure. Group and cross match of one unit of packed red cells was ordered in case a transfusion would be required.

**Fig. 2:**
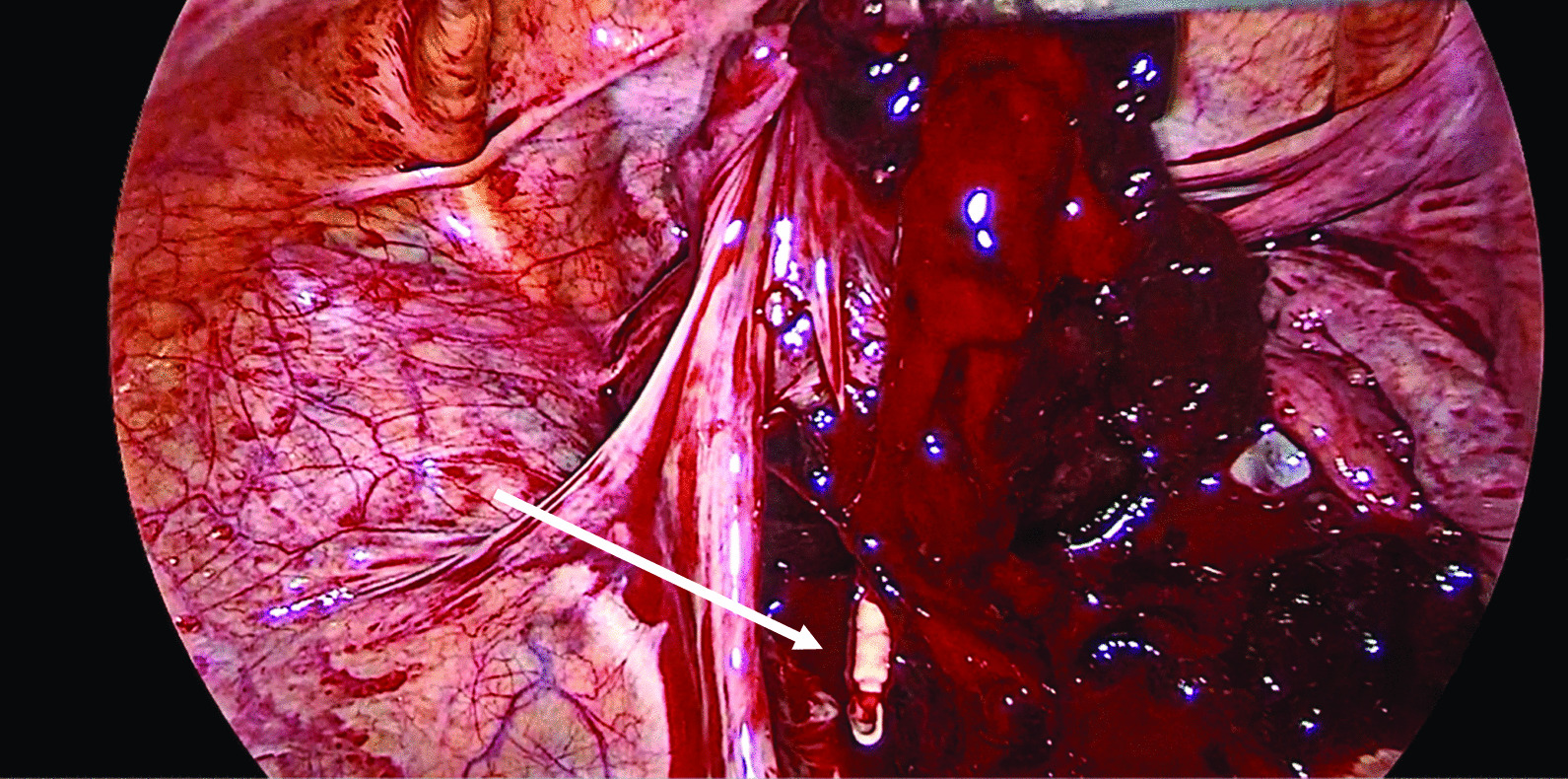
Body of the embedded LNG IUS (indicated by arrow), which is seen protruding from the left tubal pregnancy with hemoperitoneum

**Fig. 3:**
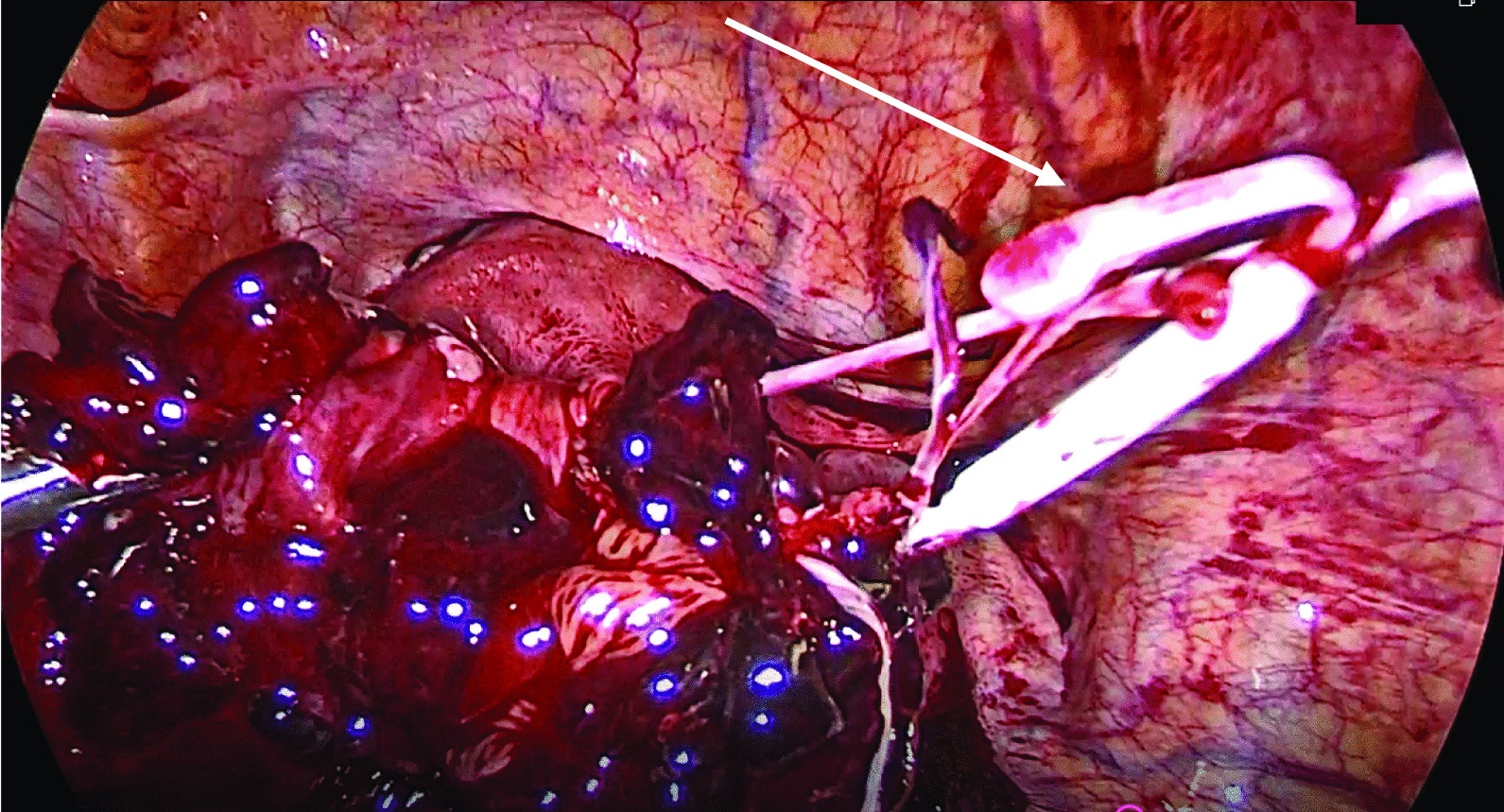
LNG IUS (indicated by arrow) being retrieved from the left tubal pregnancy

**Fig. 4:**
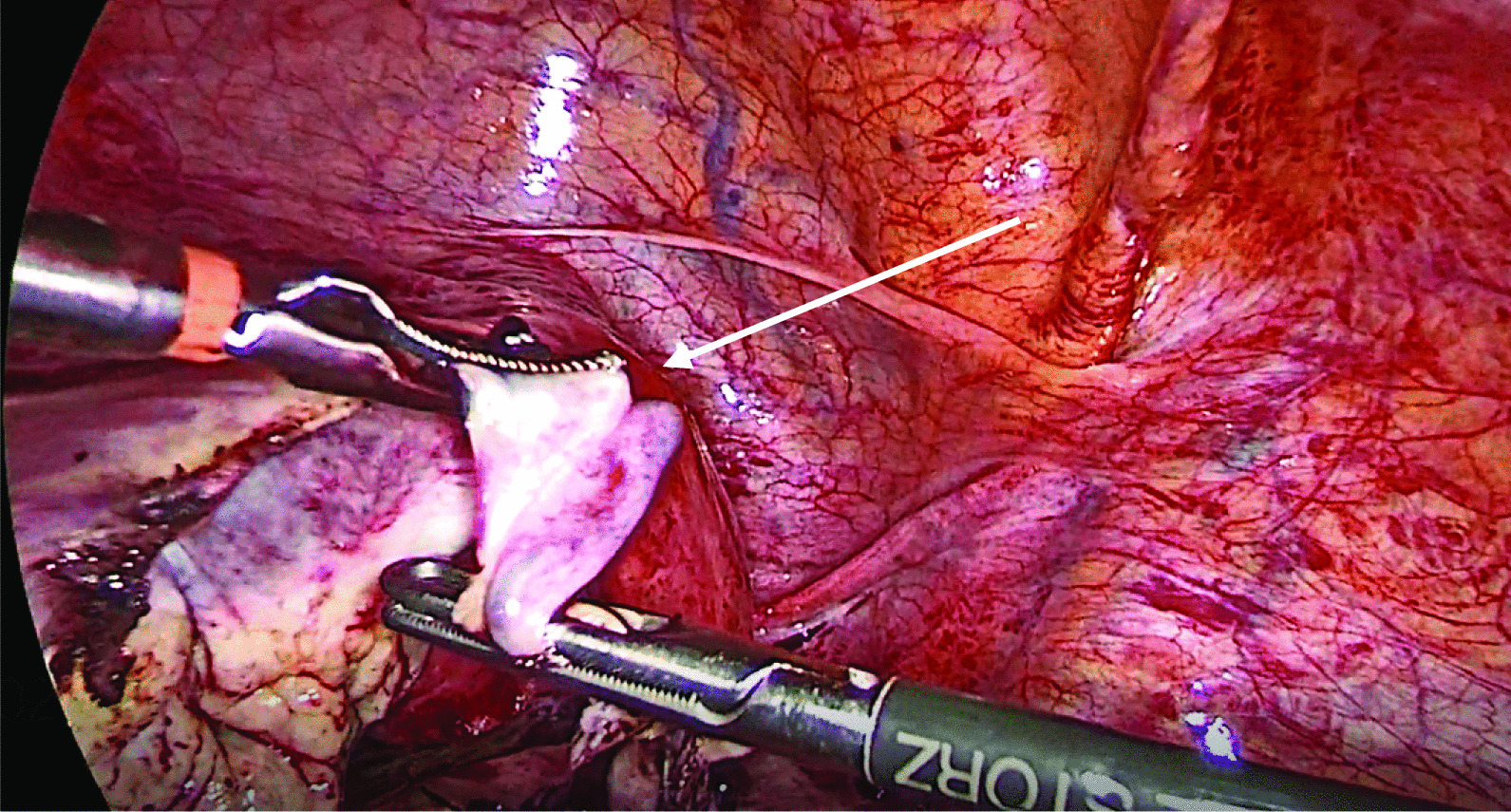
Left salpingectomy (indicated by arrow) performed using bipolar coagulation

**Fig. 5:**
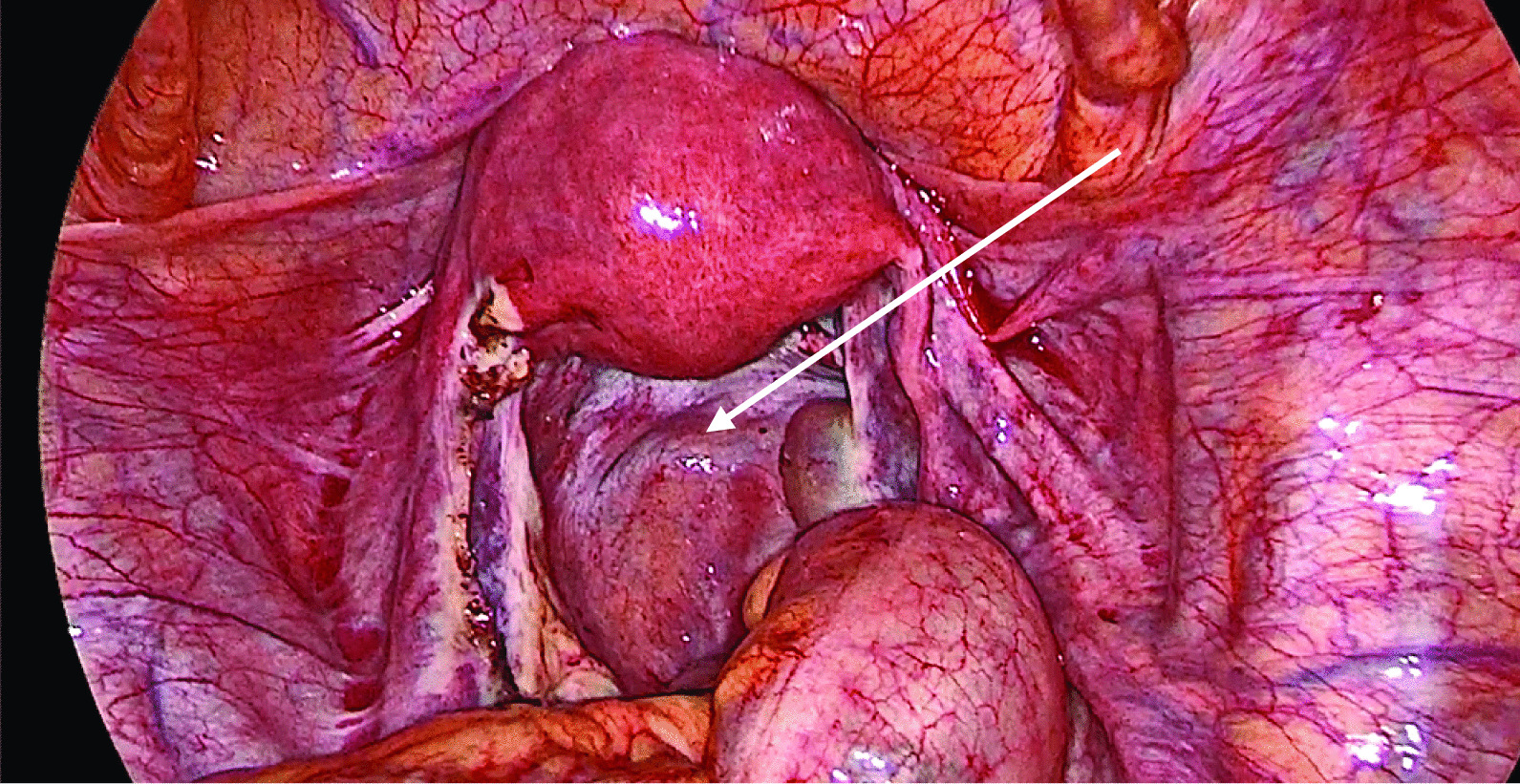
Pelvic view after completion of the procedure confirming hemostasis. Arrow points to an empty pouch of Douglas

The laparoscopy was done under general anesthesia in the Lloyd–Davis position. Cohen’s uterine manipulator was placed. Veress insufflation was performed, followed by insertion of a 10-mm primary trocar at the umbilicus. Entry and operating pressures were 20 mmHg and 15 mmHg, respectively. Two secondary ports were inserted under vision, 5 mm in the right iliac fossa and 12 mm in the left iliac fossa. On primary survey, LNG IUS was found embedded at the fimbrial end of the left fallopian tube (Fig. 2). The LNG IUS was retrieved whole under vision through the 12-mm port (Fig. 3). There was hemoperitoneum of 700 ml (Fig. 2). A ruptured left ampullary ectopic pregnancy was identified by left salpingectomy using bipolar coagulation and scissors (Fig. 4). A corpus luteum cyst was found on the right ovary with normal right fallopian tube. Suction and peritoneal lavage were performed, and hemostasis was confirmed (Fig. 5). The specimen was retrieved through the 12-mm port and taken for histology. There was no sign of uterus perforation. The pouch of Douglas and rectum appeared normal. All trocars were removed under vision.

The postoperative recovery of the patient was unremarkable. She was debriefed about the surgery and discharged the following morning. She went home on oral paracetamol and diclofenac for pain relief.

The patient was reviewed in the gynecology outpatient clinic 2 weeks later. She was asymptomatic and doing well. Histology report confirmed left ectopic tubal gestation. She reported that she desired conception. Preconception counseling was done. She was put on daily folic acid (400 µg). The patient was advised to come to the hospital as soon as she missed a period or tested positive for pregnancy for an early pregnancy ultrasound to rule out another ectopic pregnancy. Three months later she presented at the early pregnancy clinic following 5 weeks of amenorrhea. A pelvic ultrasound was done that showed intrauterine pregnancy at 5 weeks gestation.

## Discussion

The above case documents a rare occurrence of a ruptured ectopic pregnancy with the LNG IUS embedded within the affected fallopian tube.

Levonorgestrel IUS (Mirena) is a safe, reversible, and highly effective contraceptive method [2]. It is known to have other therapeutic benefits such as reduction in menstrual bleeding, anemia, and dysmenorrhea, as well as management of endometrial hyperplasia [10]. In this patient, the use of the LNG IUS was purely for contraception purposes.

Ectopic pregnancies affect approximately 2% of all pregnancies. The most common presentation of an ectopic pregnancy is abdominal pain and abnormal uterine bleeding [11]. Even though the rate of ectopic pregnancy is lower in women using LNG IUS, if a pregnancy occurs while using this IUD, there is a high risk of it being an ectopic pregnancy [2]. Current IUD use is a known risk factor for ectopic pregnancy [12]. Other risk factors for ectopic pregnancy include pelvic inflammatory disease, previous tubal surgery, previous ectopic pregnancy, and smoking [12]. On examination, features of acute abdomen due to hemoperitoneum may be present [11, 12]. The patient in this case had a presentation suggestive of ectopic pregnancy with concurrent LNG IUS use as a risk factor. However, she had other unspecific presenting features of diarrhea, vomiting, and low-grade fever. It is important to note that some patients may be asymptomatic without specific risk factors for an ectopic pregnancy [12]

On the other hand, malposition of an IUD is one of its common complications, presenting with pelvic pain and bleeding or no symptoms [13]. Although malposition is associated with reduced contraceptive efficacy, this is mostly true for copper IUDs and not LNG IUS, which has local progesterone effects [6]. Malposition is diagnosed by ultrasound [13]. However, compared with copper IUDs, the LNG IUS is more likely to be missed by ultrasonography. LNG IUS is compounded with barium sulfate, which makes it radio opaque for X-ray recognition [14]. A plain X-ray can therefore be used as an adjunctive imaging modality in the case of a lost LNG IUS not seen on ultrasound [15]. The patient in this case had a lost LNG IUS not seen on ultrasound 1 year after insertion but was lost to follow-up for additional imaging.

Uterine perforation is uncommon, with an incidence of 1 in 1000 insertions. It is a serious complication of IUD use and is often asymptomatic [16]. There is increased risk of perforation if insertion is done less than 6 months postpartum or while breastfeeding. This period is associated with endometrial atrophy with accelerated uterine involution and hence has a high risk of perforation [6, 13]. The current patient was asymptomatic for malposition or perforation for 2 years prior to the ectopic pregnancy. The transvaginal ultrasound done at diagnosis of ectopic pregnancy did not visualize the device in the left adnexa. Her LNG IUS was inserted only 2 months postpartum while breastfeeding. This may have been a risk factor for possible unrecognized perforation.

Routine transvaginal ultrasound to monitor IUD position either immediately post insertion or after 6 weeks is not recommended without clinical suspicion of malposition according to de Kroon *et al.* [17]. It has been reported that IUDs take approximately 3 months to settle into their stable position. An initially malpositioned IUD can therefore assume the correct fundal position over time [18]. In asymptomatic women with uncomplicated IUD insertion, routine ultrasound lacks benefit over clinical evaluation with string check at 6 weeks [17]. The patient in this case was asymptomatic without complications at insertion. One year later, the LNG IUS device could not be seen on clinical examination nor on ultrasound.

The mechanism of device migration is not well understood, especially in the case of tubal migration. The fallopian tube is a rare site for dislocated IUD [19, 20]. This phenomenon has been described in few case reports. There are theories from case reports about the tubal migration of an IUD. The first possibility is placement of the device at the tubal ostium during insertion with subsequent migration into the tube due to uterine contractions and tubal peristalsis [21]. The other possibility is uterine perforation with migration of the device into the peritoneal cavity and subsequent perforation of a preexisting hydrosalpinx as described by Ozdemir *et al.* in a case report [19]. The patient in this case did not have any hydrosalpinx noted intraoperatively. Perforation can be due to either immediate traumatic perforation at insertion or delayed transmural migration [22, 23]. It is also possible that, following an unrecognized perforation, there was nestling of the IUD close to the fimbrial end of the fallopian tube, with the device being enveloped within the fimbria [7, 19]. This phenomenon could have occurred antecedent to conception of the ectopic pregnancy. In the patient in this case, there was no sign of uterus perforation. The pouch of Douglas and rectum appeared normal.

The presence of an IUD within the fallopian tube is associated with an inflammatory reaction [19] interfering with tubal function and predisposing the patient to ectopic pregnancy. The tubal dysfunction is exacerbated by progesterone in the LNG IUS, which interferes with ciliary beating and tubal contractility [3]. It is possible that the embedment of LNG IUS at the fimbrial end of the fallopian tube occurred as a result of displacement from the proximal tube by the growing tubal pregnancy or its rupture.

Once an intraabdominal IUD is diagnosed, it should be removed whether it is symptomatic or not to avoid serious complications such as adhesion formation, bowel obstruction, and infertility [15, 19]. Laparoscopy is the surgical approach of choice as it is safe and effective. It provides good visualization to locate and remove a lost IUD [15]. In this case, the diagnosis of the LNG IUS within the fallopian tube and its removal were done laparoscopically.

## Conclusion

Migration of the LNG IUS into the fallopian tube is a rare occurrence that is not well understood. In the case presented, a LNG IUS was found embedded within the fimbrial end of the fallopian tube that had a ruptured ectopic pregnancy. Surgical treatment with laparoscopy is recommended for intraabdominal IUD to prevent complications. The patient made a good recovery and proceeded to have an intrauterine pregnancy 3 months later, as desired.

## Data Availability

Clinical data and complementary examinations are available from the corresponding author on reasonable request.

## Abbreviations

IUD: Intrauterine device
LNG IUS: Levonorgestrel intrauterine system
